# Monitoring social distancing under various low light conditions with deep learning and a single motionless time of flight camera

**DOI:** 10.1371/journal.pone.0247440

**Published:** 2021-02-25

**Authors:** Adina Rahim, Ayesha Maqbool, Tauseef Rana

**Affiliations:** Department of Computer Software Engineering, NUST, Islamabad, Pakistan; Wuhan University of Science and Technology, CHINA

## Abstract

The purpose of this work is to provide an effective social distance monitoring solution in low light environments in a pandemic situation. The raging coronavirus disease 2019 (COVID-19) caused by the SARS-CoV-2 virus has brought a global crisis with its deadly spread all over the world. In the absence of an effective treatment and vaccine the efforts to control this pandemic strictly rely on personal preventive actions, e.g., handwashing, face mask usage, environmental cleaning, and most importantly on social distancing which is the only expedient approach to cope with this situation. Low light environments can become a problem in the spread of disease because of people’s night gatherings. Especially, in summers when the global temperature is at its peak, the situation can become more critical. Mostly, in cities where people have congested homes and no proper air cross-system is available. So, they find ways to get out of their homes with their families during the night to take fresh air. In such a situation, it is necessary to take effective measures to monitor the safety distance criteria to avoid more positive cases and to control the death toll. In this paper, a deep learning-based solution is proposed for the above-stated problem. The proposed framework utilizes the you only look once v4 (YOLO v4) model for real-time object detection and the social distance measuring approach is introduced with a single motionless time of flight (ToF) camera. The risk factor is indicated based on the calculated distance and safety distance violations are highlighted. Experimental results show that the proposed model exhibits good performance with 97.84% mean average precision (mAP) score and the observed mean absolute error (MAE) between actual and measured social distance values is 1.01 cm.

## Introduction

COVID-19 belongs to the family of coronavirus caused diseases, firstly reported in Wuhan, China at the end of December 2020. China has announced its first death from the virus on January 11, a 61 years old man. On March 11, World Health Organization (WHO) [1, 2] declared it pandemic due to its spread over 114 countries with a death toll of 4000 and active cases of 118000 [3]. Data from Johns Hopkins University showed that more than seven million people were confirmed to have the coronavirus with at least 406,900 dying from the disease on June 8. Several health organizations, scientists, and doctors tried to develop vaccine but no success is observed so far. This situation forces the world to find out an alternative solution to avoid drastic results. Lockdown was imposed globally and maintaining safe social distance is reported to be the alternate solution to cope with this drastic situation. The term social distancing is the best idea in the regulation of efforts made to minimize the spread of COVID-19 [4]. The basic objective is to reduce the physical contact between the infected and the healthy people. As prescribed by WHO, people should maintain at least 1 meter (m) distance from each other to control the spread of this disease [1, 5, 6].

This paper aims to mitigate the effects of coronavirus disease along with minimum loss of resources; this disease has badly impacted the global economy. Secondly, to provide a highly accurate solution for the detection of people to help out in monitoring social distancing during the night. Especially, in summer when the heat is at its peak, people having congested homes find ways to get out of their homes during the night with their families to take fresh air. During this serious situation, it is necessary to take proper action. Recently, Eksin et al. [7] evaluated the susceptible infected recovered (SIR) model where they included a social distancing term. They showed that the spread of disease depends upon people’s social behavior. They assessed the results of the SIR model with and without behavior change factor and found that a simple SIR model did not get well performance even after many repeated observations; whereas, their updated SIR model with behavior change factor showed good results and corrected the initial error rate. In a similar context, a landing AI [8] company has declared the development of an AI tool for monitoring social distance in the working area. In a short report [8], the firm professed that the prospective tool will be able to observe people, whether they are following safety distance criteria by examining real-time video streams captured by the camera. They affirmed that this tool can be easily combined with available security cameras in different working areas to ensure a safe distance between workers. The world-leading research company Gartner Inc. [9] declared landing AI as cool vendors in AI core technologies to acknowledge their timely incentive to support the fight against the deadly situation of COVID-19 [10].

In this article, a deep learning-based solution is proposed for the automatic detection of people and monitoring social distance in low light environments. The first contribution of this article is the performance evaluation of YOLO v4 on low light conditions without applying any image cleansing approaches. As in past low light environments are not much focused, few have focused the problem but only in the context of enhancing low light scenarios and improving visibility [11–14]; whereas, in the real-time object detection and monitoring, this approach is not feasible because it takes more time to enhance low light scenarios at first place and then apply object detection techniques. So, the real-time application should have to give a timely response with high accuracy. Secondly, a social distance monitoring solution is proposed by considering precise speed-accuracy tradeoff and is evaluated on our custom dataset. From experimental results, it is observed that the model exhibited good performance with a balanced mAP score and MAE [15] of 1.01 cm.

## Related work

In this section, we briefly introduce previous work done on the social distancing in the context of the 2019 novel coronavirus disease. As the disease spread at the end of December, researchers started work to pay their contributions in the deadly situation. Social distancing was suggested as the alternative solution. The different research studies were conducted to provide an effective social distancing solution. In the same background, Prem et al. [16] studied the consequences of social distancing measures on the progression of the COVID-19 epidemic in Wuhan, China. They used synthetic location-specific contact patterns to imitate an ongoing trajectory outbreak using age structure susceptible-exposed-infected removed (SEIR) models for several social distancing measures. They interpreted that a sudden rise in interventions will lead to an early secondary peak but it will flatten gradually with time. As we all can understand social distancing is important to cope with the current situation but economically it is a drastic measure to flatten the curve against infectious diseases. Adolph et al. [17] emphasized the situation of USA where they gathered state-level responses regarding social distancing and found the contradiction in the decision among policymakers and politicians which causes a delay in imposing the social distancing strategies resulting in ongoing harm to public health. On the brighter side, social distancing helped a lot to control the spread of disease but it has also affected economic productivity. In the same background, Kylie et al. [18] have studied the association between transmissibility and social distancing and found that association decreases as transmissibility decreases within different provinces of China. According to the study, the intermediate level of activity could be allowed while avoiding an immense outbreak.

Since the COVID-19 pandemic began, many countries are seeking for technology-oriented solutions. Asian countries have used a range of technologies to fight against COVID-19. The most used technology is tracking location by phones where the data of COVID-19 positive people are saved, based on this data their near about healthy people are monitored. Germany and Italy are using anonymized location data to monitor lockdown. UK has launched an application (app) named C9 corona symptom tracker [19] that helps people to report their symptoms. Similarly, South Korea launched an app named Corona 100m [19] that has stored the location of infected people and generate alert to healthy people when they came near to corona patients at a distance of 100m. India has developed an app that helps people to maintain a specific distance from a person who has tested corona positive. Besides this, India, South Korea, and Singapore are taking benefit from CCTV footage [19] to monitor the recently visited places of COVID-19 patients to track down the infected people. China is utilizing AI-powered thermal cameras [19] to identify those people in the crowd having the temperature. Such inventions in this drastic situation might help to flatten the curve but at the same time, it results in a threat to the personal information.

Object detection helped a lot in this deadly situation. Many of the researchers have investigated the situation [20–23] to detect various types of objects to help out the scenario. Human detection [24–27] is an established area of research. Recent advancements in this field [28, 29] had created the demand for intelligent systems to monitor unusual human activities. Despite the fact, human detection is an interesting field because of many reasons like faint videos, diverse articulated pose, background complexities, and limited machine learning capabilities; hence, existing knowledge can boost the detection performance [20]. Narinder et al. [21] motivated by the notion of social distancing proposed a deep learning-based structure to automate the task of observing social distance using surveillance video [22]. They used YOLO v3 [30] algorithm with a deep-sort technique for the separation of people from the background and tracking of detected people with the help of bounding boxes. Cob et al. [23] investigated the relation of COVID-19 growth rates in US with shelter in place orders (SIP). They presented a random forest machine learning model for their predictions and found the SIP orders very effective. Their study showed that SIP orders will not only be helpful for the US but also will help highly populated countries to reduce the COVID-19 growth rate. Deep learning is the popular area to perform object detection which gained a huge interest in the modern research field. Deep learning techniques have successfully applied in the drastic situation of COVID-19 by automating the task of face mask detection [31], detection of COVID-19 cases with X-ray images [32], lung infection measurement in CT images [33], COVID-19 patients monitoring [34] and most importantly monitoring social distancing [20–23].

Different research studies were conducted to provide a better and effective social distance monitoring solution as we discussed above but no one has focused on the low light environments. Besides this, we have not found any real-world unit distance mapping solution. To fillup this research gap, this article mainly focuses on low light conditions and to come up with a real-world unit distance mapping strategy that simplifies social distance monitoring tasks to help out in this deadly situation.

## Background of deep learning models

Several deep learning algorithms are available and every newly developed algorithm has resolved the problems of the previous one in some way. Conventional object detection algorithms use classifier based procedure, where the classifier runs on a slice of the image in sliding window fashion, this is how Deformable Parts Model (DPM) [35] works. In R-CNN ancestry (R-CNN [36], Fast R-CNN [37] and Faster R-CNN [38]) classifier run on region proposals that are considered as bounding boxes. These algorithms exhibit good performance, especially Faster R-CNN with an accuracy of 73.2% mAP, but because of their intricate pipeline, they show poor performance in the context of speed with 7 frames per second (FPS), which limit them for real-time object detection.

This is where YOLO fits, a real-time object detection system with a creative perspective of reviewing object detection as a regression problem was introduced in 2016 by Joseph et al. [39]. YOLO exhibits good performance as compared to previous region-based algorithms in terms of speed with 45 FPS by maintaining good detection accuracy of 63.4% mAP. Despite good speed and performance, YOLO made notable localization errors. Moreover, YOLO has low recall. To resolve the shortcomings of YOLO, in the same year authors of YOLO released YOLO second version where recall and localization were mainly focused without affecting classification accuracy. YOLO v2 [40] gained a speed of 67 FPS and mAP reached 76.8%. YOLO v2 is also called YOLO 9000 because of its ability to detect objects of more than 20 classes by mutually optimizing classification and detection. The YOLO v3 [30] developed in 2018 brought new improvements in speed and accuracy, but the main idea remained the same.

Recently YOLO v4 is released by Alexey et al. [41]. In comparison with its direct predecessor YOLO v3, average precision (AP) and FPS increased by 10 to 12 percent. In experiments on the MS COCO [42] dataset, it obtained 43.5% AP score and achieved a real-time speed of approximately 65 FPS on Tesla V100, vanquishing over the most accurate and fastest detectors in terms of both accuracy and speed. Most of the detectors require multiple GPUs for training with a large batch size; whereas, training on a single GPU makes the training process very slow. YOLO v4 resolved this issue by presenting a fast and accurate object detector that can be trained with a smaller batch size on a single GPU. Below we have briefly described the architecture of general object detectors and the newly introduced YOLO v4 model.

### General architecture of object detector

Ordinary object detectors like R-CNN, Fast R-CNN, and Faster R-CNN are two-stage detectors made up of three parts: backbone, neck, and head.

- **Backbone**: Models like VGG [43], DenseNet [44], and ResNet [45] are used as feature extractors, first trained on image classification dataset, and then fine-tuned on detection dataset. These networks construct different levels of features which will result in a deeper network and be useful for prior parts of object detection networks.
- **Neck**: Extra layers lie between backbone and head which will be helpful for feature map extraction from previous backbone stages. Different feature map extraction techniques are used, e.g., YOLO v3 uses Feature Pyramid Network (FPN) [46] for extraction of feature maps of different scales from the backbone, where every next layer gets in input the merged results of previous layers and produces different levels of the pyramid. Classification/ regression (head) is applied on every pyramid level which helps in the detection of different sizes of objects.
- **Head**: This is responsible for assigning a class to objects and generating bounding boxes around it (classification and regression). One stage detectors like YOLO apply classification/regression to each anchor box.

### YOLO v4 architecture

**Fig 1:**
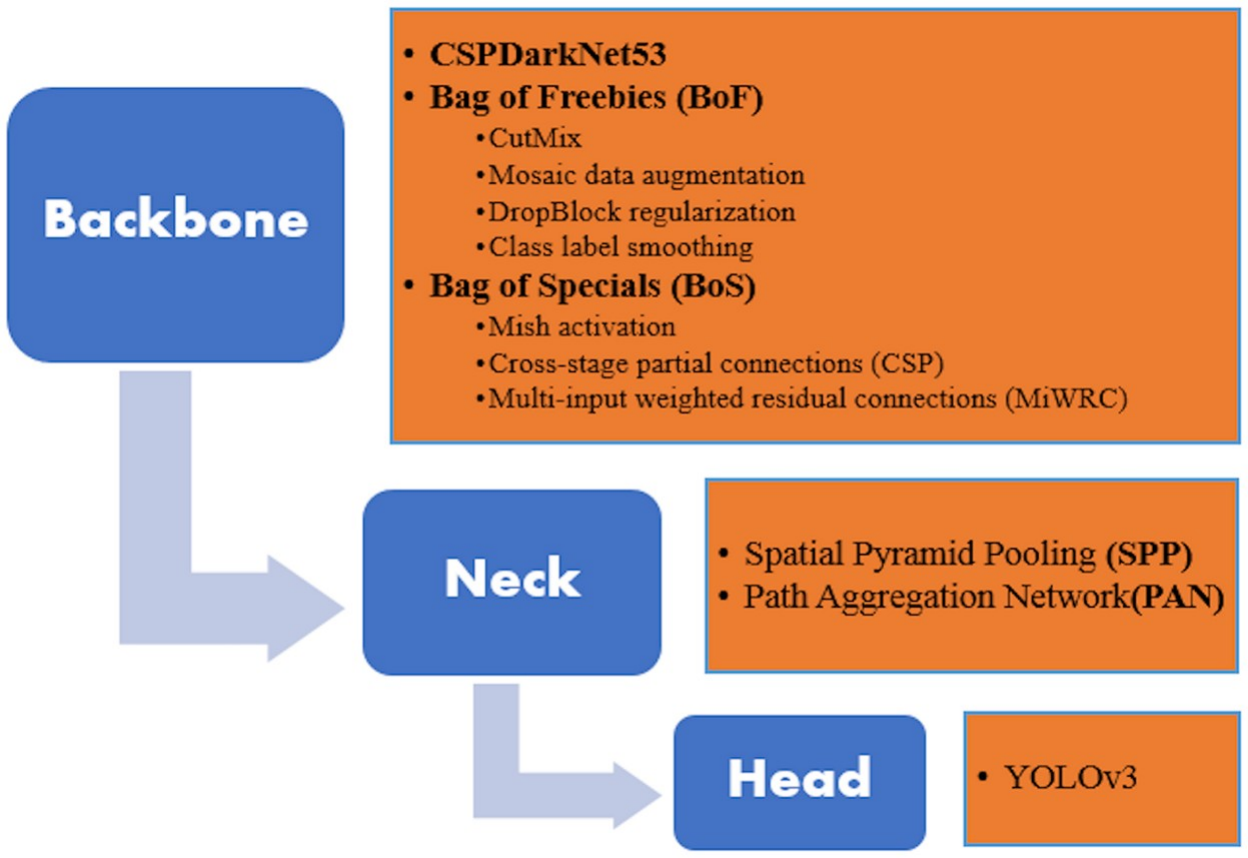
Schematic representation of YOLO v4 architecture.

In this section, we discuss YOLO v4. Fig 1 shows a diagrammatic representation of YOLO v4 architecture.

- **Backbone**: It employs CSPDarknet53 as a feature extractor with a graphics processing unit (GPU). Few backbones are more appropriate for classification than for detection. For example, CSPResNext50 is better than CSPDarknet53 for image classification; whereas, CSPDarknet53 is proved better in terms of object detection. For better detection of small objects, the backbone model needs a higher network size as an input and for higher receptive fields more layers are required.
- **Neck**: For feature map extraction, it uses Path Aggregation Network (PAN) and Spatial Pyramid Pooling (SPP). PAN used in YOLO v4 is the modified version of the original PAN where addition is replaced with concatenation. In the original version after minimizing N4 size to get the same spatial size of P5, they summed this new depleted N4 with P5. This reoccurs at all layers of Pi+1 to create Ni+1. In YOLO v4 rather than adding Ni with each Pi+1, they concatenated them. If we glass over SPP it mainly does max-pooling over 19 × 19 × 512 feature map distinct kernel sizes k = 5, 9, 13 with the same padding to keep the spatial size same. Four feature maps are merged to form 19 × 19 × 2048 magnitude. This increases the neck receptive field with improvement in the model’s accuracy and minimal rise of inference time.
- **Head**: YOLO v4 utilizes the same head as YOLO v3 with the anchor-based detection steps.

#### YOLO v4 performance optimization

The authors of YOLO v4 differentiated between two types of methods that are used to improve object detector’s accuracy. They examined both types of methods to obtain fast operating speed with high accuracy. Both types are as follows:

- **Bag of Freebies (BoF)**: Procedure that produces an object detector that delivers better accuracy without increasing inference cost. One of its examples is data augmentation, the model trained on small datasets has poor generalization ability which leads these models towards overfitting. Overfitting is the problem that usually arises when a deep neural network tries to learn the most frequently occurring pattern. As several methods were proposed to resolve the problem of overfitting. Data augmentation [47] is from one of those methods, by utilizing it we can reduce overfitting on the models. Many data augmentation techniques are available like brightness alteration, disparity, noise, and saturation or we can do geometric twisting like cropping, rotating, and flipping. Some other bag of freebies include regularization approaches to avoid overfitting. Conventional regression technique is mean squared error (MSE) [48], the mean of the sum of squared difference between observed and true values as described in Eq (1).
  $$\begin{array}{c} MSE=\left(\frac{1}{n}\right)\sum_{i=1}^{n}{\left(y_{i}-x_{i}\right)}^{2} \end{array}$$ (1)
  MSE treats variables as self-sufficient rather than unified. To surpass this, IoU [49] loss is proposed, which takes into account the area of the ground truth bounding box and predicted bounding boxes (BBox). This notion is further enhanced by GIoU [50] loss by adding orientation and shape of an object with the area. Besides GIoU, CIoU is introduced which takes into account overlaying area, aspect ratio, and distance between center points. YOLO v4 uses CIoU loss for bounding boxes, because of its good performance and faster convergence.
- **Bag of Specials (BoS)**: Those elements and post-preprocessing techniques that only increase a small amount of inference cost but bring notable improvement in the object’s detection accuracy. YOLO v4 considers the modified Spatial Attention Module (SAM) [51]. In SAM instead of using max and average pooling, the feature map is passed through a convolutional layer with a sigmoid activation function and then multiplied to the original feature map. YOLO v4 uses the Mish activation function in the backbone as described in Eq (2). E.g., using Mish with Squeeze Excite Network [52] on the CIFAR100 dataset improves accuracy by 0.494% and 1.671% in comparison with the same network where ReLU and Swish were used [53].
  $$\begin{array}{c} f\left(x\right)=xtanh(ln\left(1+e^{x}\right)) \end{array}$$ (2)

## Materials and methods

### Training dataset

**Fig 2:**
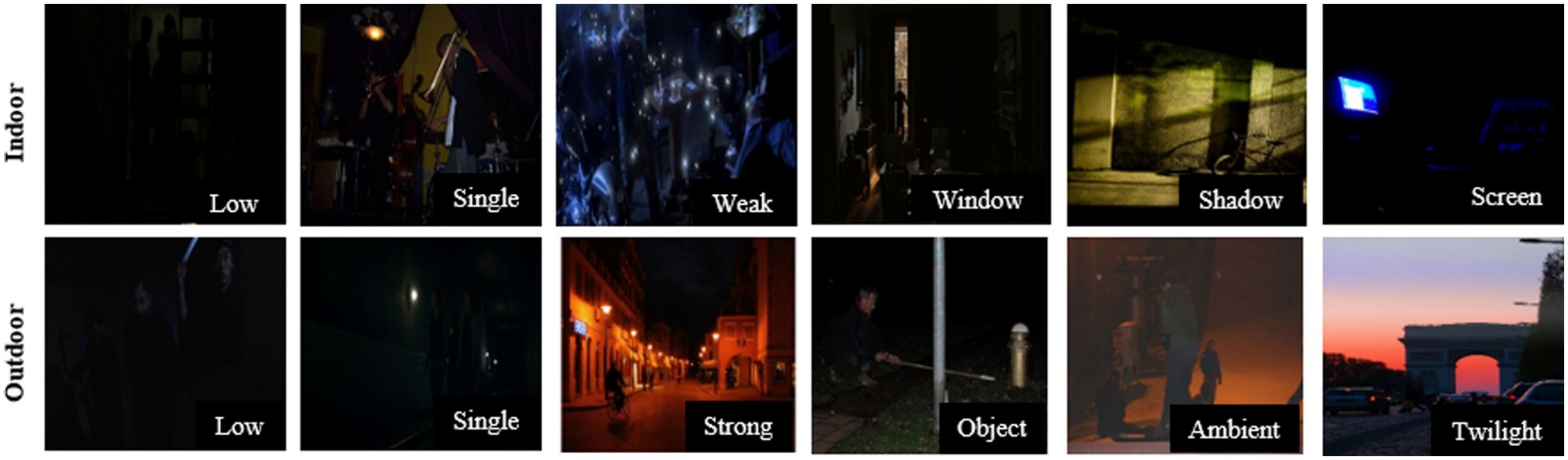
Example of low-light image types in the ExDARK dataset.

In this paper, to tune up the object detection model for human detection under various low light conditions, a recently released ExDARK dataset [54] is considered which specifically focuses on a low-light environment. In this dataset, 12 different classes of objects are labeled, out of which we fetched data of our desired class for training. This dataset contains different indoor and outdoor low light images; furthermore, the data is subdivided for low light environment into 10 classes ambient, object, strong, twilight, low, weak, screen, window, shadow, and single. Sample images of various indoor-outdoor low-light environments from the dataset are shown in Fig 2.

### Testing dataset

**Fig 3:**
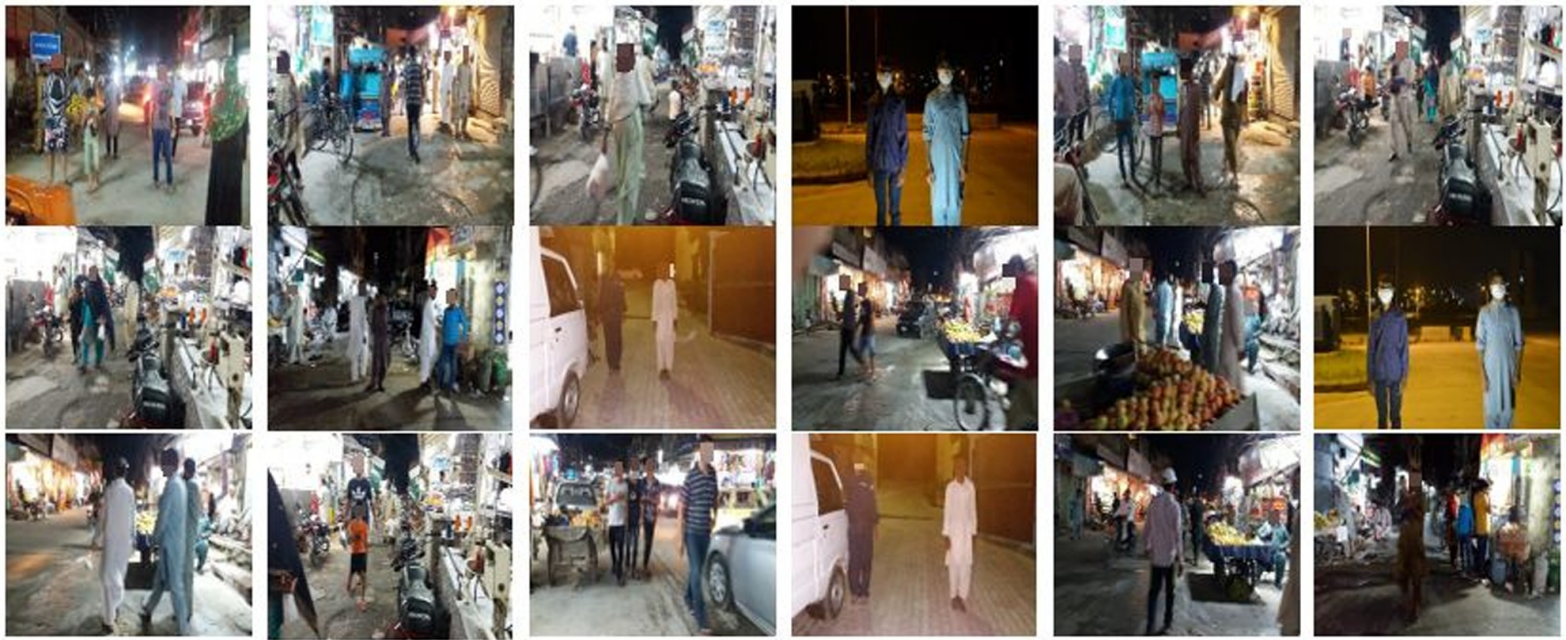
Custom dataset for testing.

A custom dataset is used for the evaluation of the proposed model. The dataset is collected from the market of Rawalpindi, Pakistan during the night in the days of COVID-19. Pakistan is one of the most urbanized countries in South Asia with a 3% yearly urban population growth rate. The large population and congested streets make it a riskier place in the growth of COVID-19 and it is very difficult to maintain safety distance in such narrow places. Hence, the monitoring system should need to have high accuracy in terms of the detection and location of the people. Evaluation of the proposed framework in such a highly-populated area will help us to better analyze the performance of the model. Test dataset is the collection of 346 RGB frames. Frames are collected with motionless ToF camera of Samsung galaxy note 10+ installed 4.5 feet above the ground where a 0° regular camera view calibration is adopted. Sample images of low-light conditions from the custom dataset are shown in Fig 3.

### Monitoring social distancing with deep learning and a single motionless time of flight (ToF) camera

The emergence of deep learning has caught much attention and became a presiding technology that introduced a variety of techniques to solve different challenges including self-driving [55], fraud detection [56–58], robotics [59], language translations [60], medical diagnosis [61], and many more [62]. Most of these challenges revolve around object detection, classification, segmentation, recognition, and tracking, etc.

In this research article, a deep learning-based solution is proposed that uses an object detection model for automating the task of social distance monitoring at fixed camera distance (*C*_*d*_) under various low light environments. To monitor social distance at *C*_*d*_ motionless ToF [63] camera is utilized along with the YOLO v4 algorithm to maintain speed-accuracy tradeoff.

ToF cameras give real-time distance images which simplify human monitoring tasks. These cameras utilize light pulses. The light of the camera is switched on for a short time interval and the resultant light pulse brightens the scene and comes back by striking the object. This reflected light encounters a reflection delay depending on the distance of the object. The camera lenses assemble the incoming light and create an image on the sensor. ToF camera to object distance is calculated by Eq (3).

$$\begin{array}{c} C_{d}=\frac{1}{2}\times S_{L}\times L_{p}\times \frac{S_{2}}{S_{1}+S_{2}} \end{array}$$ (3)

Where *S*_*L*_ is the speed of light, *L*_*p*_ is the length of the pulse, *S*_1_ is gathered charge when light is emitted and *S*_2_ represents the charge when there is no light emission. The view *V* captured by ToF camera is the three tuple value V = (*F*, *T*_*D*_, *C*_*p*_), where *F* is an RGB frame with height and width, *T*_*D*_ is a safe distance threshold value, and *C*_*p*_ shows camera position in real world environment. In a given *V* we are eager to find number of people *p*_*o*_ = (*p*_1_, *p*_2_, *p*_3_, …, *p*_*n*_) and their self-distance $PD=(ED_{p1,p2},ED_{p1,p3},..,ED_{p1,p_{n}},ED_{p2,p3},ED_{p2,p4},..,ED_{p2,p_{n}},\ldots ,ED_{p_{n-1},p_{n}})$ where *ED* ∈ ℜ_+_ and *p*_*n*_ is overall people detected in one frame. We are also keen to find the value of safety threshold *T*_*D*_ to monitor safety distance violations (*PD* < *T*_*D*_|*PD* = *T*_*D*_|*PD* > *T*_*D*_).

### People detection in *F* by deep learning

For the detection of objects, the YOLO v4 model is trained on the ExDARK dataset. We trained our model on two different network sizes (320 × 320 and 416 × 416) and evaluate the performance in both cases. The model trained on 416 × 416 network size shows the highest mAP value as shown in Table 1. The trained model *T*_*m*_ = (*BB*_*i*_, *CL*_*i*_, *CS*_*i*_) is tuple of three values, where *BB*_*i*_ shows bounding boxes coordinates of detected *p*_*o*_ in *F*, *BB*_*i*_ = (*Xmin*_*i*_, *Ymin*_*i*_, *Xmax*_*i*_, *Ymax*_*i*_), class labels *CL*_*i*_, and confidence score *CS*_*i*_, ∀*i* ∈ {1, 2, 3, …, *n*}. We have created list of all center points *CP*_*i*_ of detected *BB*_*i*_ in *F*, *CP*_*i*_ = {(*x*1, *y*1), (*x*2, *y*2), …, (*x*_*n*_, *y*_*n*_)}.

**Table 1:** Model’s performance evaluation using COCO detection metrics at different IoU threshold.

**Backbone**	**FPS**	**Network Size**	**TP**	**FP**	**FN**	**Prec.**	**Rec.**	**F1-score**	**mAP**	**Total BFLOPS**
CSPDarknet-53	46.2 (T)	416 × 416	382	37	13	0.91	0.97	0.94	97.84%	59.563
	52.5(T)	320 × 320	257	64	38	0.85	0.90	0.88	92.68%	35.244
**Backbone**	**FPS**	**Network Size**	**TP**	**FP**	**FN**	**Prec.**	**Rec.**	**F1-score**	**mAP**	**Total BFLOPS**
CSPDarknet-53	-	416 × 416	345	74	50	0.82	0.87	0.85	86.67%	59.563
	-	320 × 320	273	148	122	0.65	0.69	0.67	56.37%	35.244
**Backbone**	**FPS**	**Network Size**	**TP**	**FP**	**FN**	**Prec.**	**Rec.**	**F1-score**	**mAP**	**Total BFLOPS**
CSPDarknet-53	-	416 × 416	285	134	110	0.68	0.72	0.70	68.17%	59.563
	-	320 × 320	237	184	158	0.56	0.60	0.58	53.93%	35.244

### Specifying *T*_*D*_ in *F*

**Fig 4:**
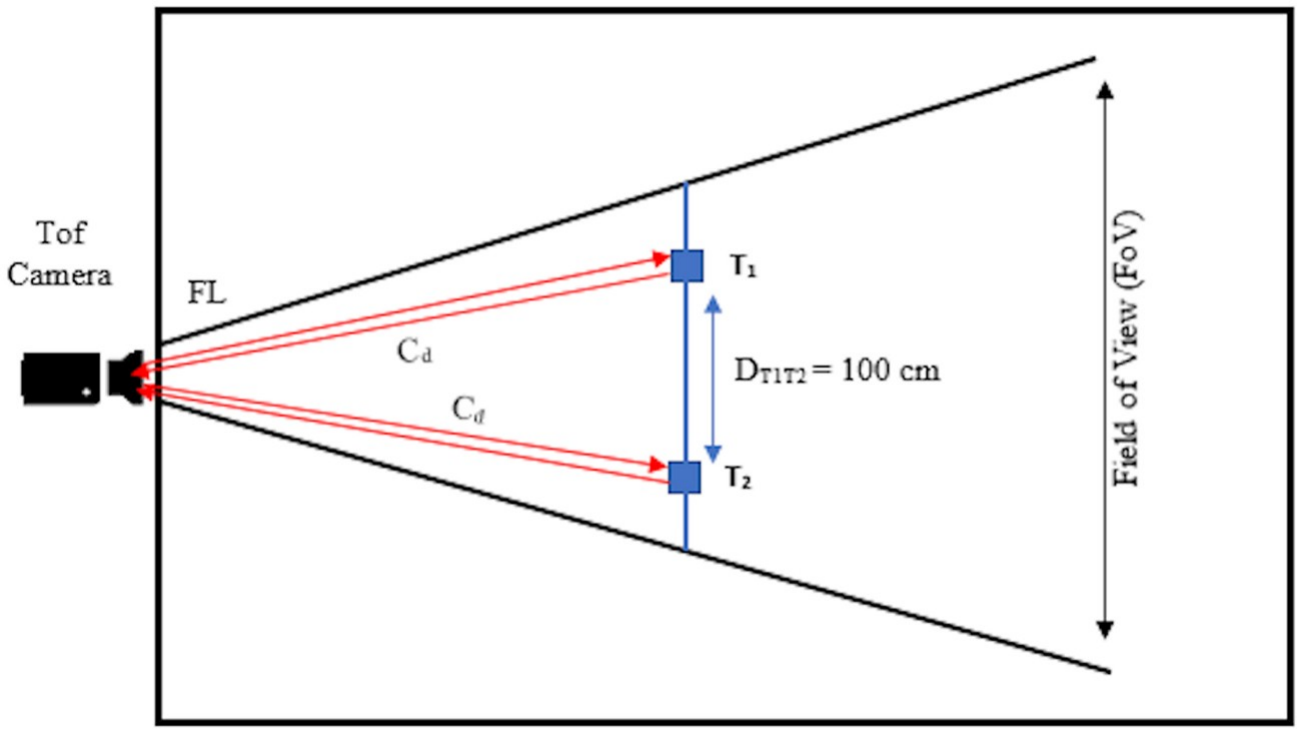
Environmental setup of motionless ToF camera based social distance monitoring at fixed camera distance *C*_*d*_ where *T*1 and *T*2 are target objects placed in environment to initialize monitoring process.

The considered safety threshold value to control the spread of disease is 100 cm as specified by WHO [1]. For initializing the monitoring process we have placed two temporary targets (*T*1, *T*2) in the real-world environment with the actual self distance *D*_*T*1*T*2_ of 100 cm at *C*_*d*_ and capture image. The captured image is passed to *T*_*m*_ and calculated Euclidean distance *E*_*d*_ between *CP*_*i*_ of detected bounding boxes by Eq (4). The calculated *E*_*d*_ gives us distance between *T*1 and *T*2 in *F* in the form of pixels which is equivalent to real-world unit distance *D*_*T*1*T*2_. This *E*_*d*_ will be used as a threshold value to filter newly coming people in the *V*. The environmental arrangement of ToF camera with target objects T1, T2, and safety threshold distance *D*_*T*1*T*2_ is shown in Fig 4.

$$\begin{array}{c} \begin{array}{c} E_{d}=\sqrt{{\left(x_{T2}-x_{T1}\right)}^{2}+{\left(y_{T2}-y_{T1}\right)}^{2}}\\ T_{D}=E_{d} \end{array} \end{array}$$ (4)

### Pixels to real-world unit distance mapping

To convert *T*_*D*_ from pixel distance to unit distance (cm) we found that *T*_*D*_ is directly proportional to *D*_*T*1*T*2_ as described in Eq (5).

$$\begin{array}{c} \begin{array}{c} D_{T1T2}\propto T_{D}\\ D_{T1T2}=k\times T_{D}\\ k=\frac{D_{T1T2}}{T_{D}} \end{array} \end{array}$$ (5)

Here *k* is the constant which represents one pixel which is equivalent to $\frac{D_{T1T2}}{T_{D}}$ units. We convert the distance between the center points of newly coming objects at *C*_*d*_ in *V* into units by Eq (6).

$$\begin{array}{c} Du_{i}=k\times PD \end{array}$$ (6)

**Fig 5:**
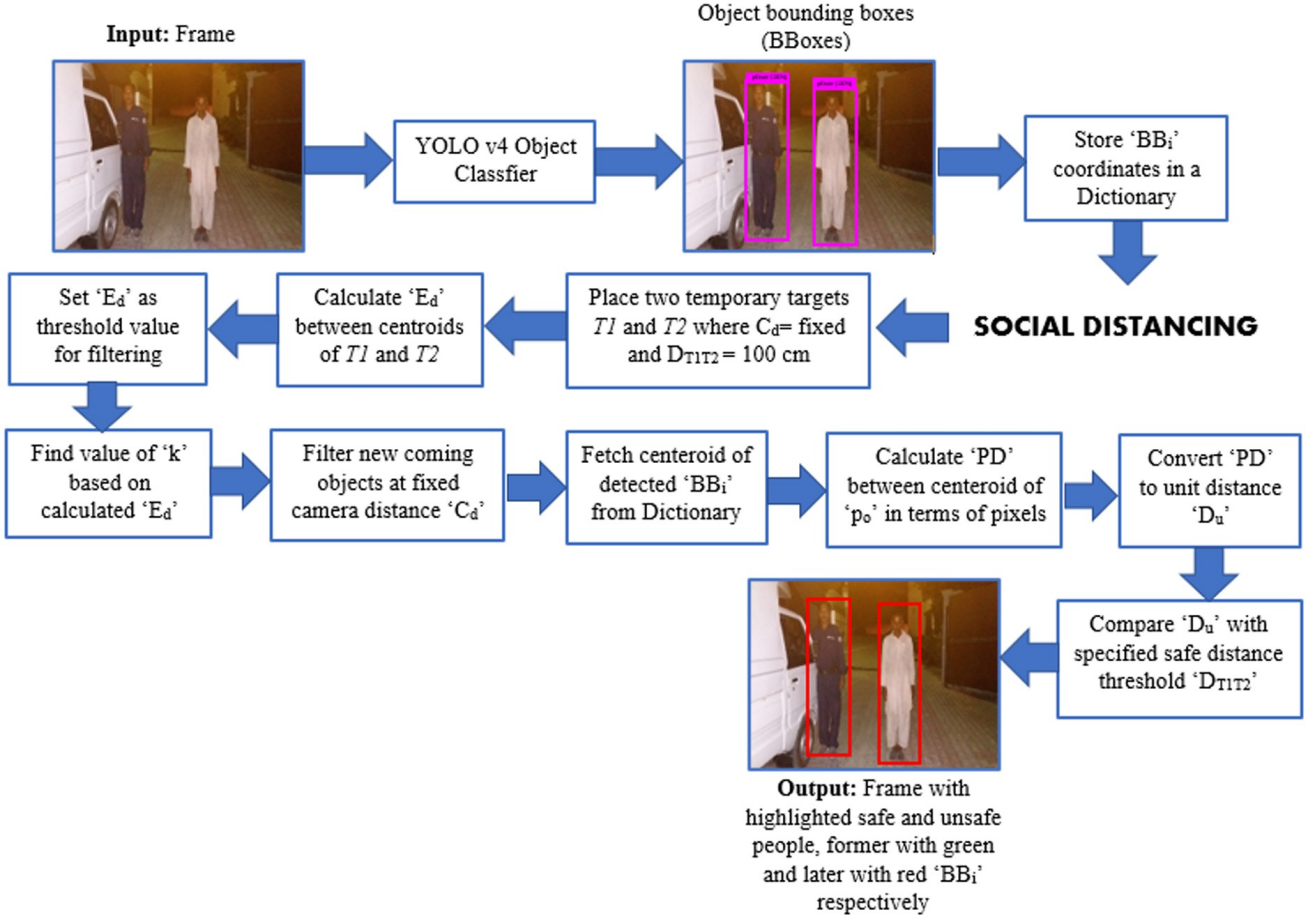
Workflow model.

Where *Du*_*i*_ is measured distance in units, *k* is constant which stores pixel to unit equivalent value, and *PD* is the Euclidean distance between the *CP*_*i*_ of all detected persons in *F*. The workflow of the proposed model is shown in Fig 5.

## Experiments & results

### Experimental setup

In the ExDARK image classification experiment, the selection of hypermeters are as follows: training steps are 35000 and 50000 at two different network sizes 320 and 416; batch size and subdivisions are 64 and 16; the polynomial decay learning rate scheduling strategy is adopted with an initial learning rate 0.001; the warm-up steps are 1000; Momentum and weight decay of 0.949 and 0.0005 respectively. From a bag of freebies (BoF) mosaic data augmentation technique is utilized. From the bag of specials (BoS) mish and leaky- ReLU [64] activation functions are used. The network size is 320 × 320 and 416 × 416 with 3 channels and the initialized IoU threshold for ground truth allocation is 0.213. The IoU normalizer is 0.07 and CIoU loss is used for bounding boxes. To cut off a large number of rectangular boxes and choose the best one greedy non-maximum suppression (NMS) is used. The experiments are done on Tesla T4 GPU with 16 GB memory, CUDA v10010, and cuDNN v7.6.5.

### Evaluation standards

Common evaluation indicators for object detectors are Precision, Recall, and AP. The subsequent explains the purpose of these indicators in the context of person detection under various low light conditions. Precision shows how accurately the model has predicted the people. Recall is described as the number of truly detected people over the sum of truly detected people and undetected people in the image. AP is the mean of the precision score after every true object is detected as shown in Eq (7). It comprehends the performance of the object detection algorithms. Having extensive assessment ability AP is used as an assessment indicator in this research which is equivalent to mAP in COCO detection metrics [42].

$$\begin{array}{c} AP=\frac{\sum_{n}precision}{n} \end{array}$$ (7)

### Performance evaluation

**Fig 6:**
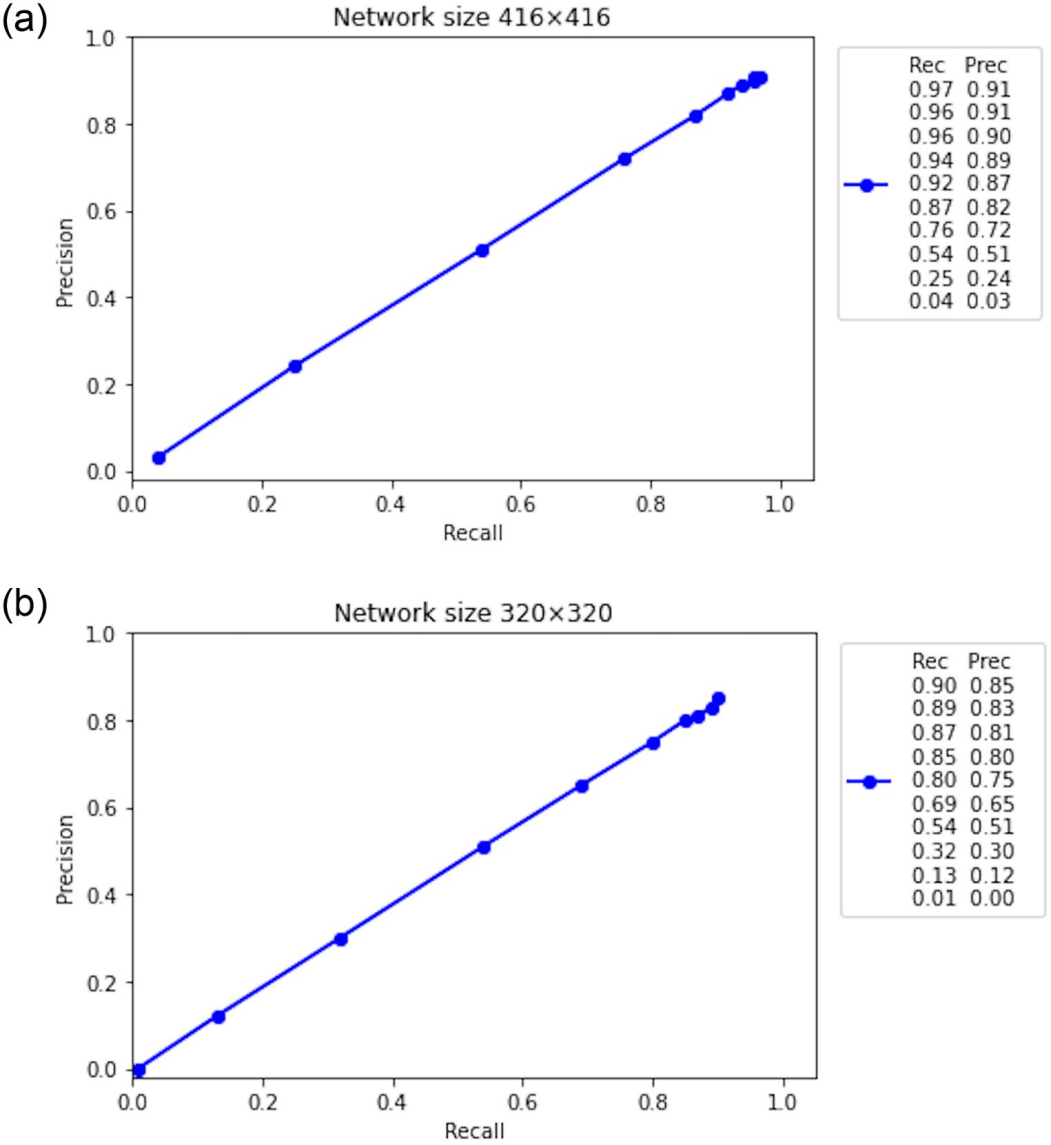
COCO evaluation, the IoU threshold ranges from 0.5 to 0.95 with a step size of 0.05.

By performing a series of experiments, we evaluate the performance of the trained model by COCO detection metrics. Table 1 shows precision (Prec), recall (Rec), F1-score, false positives (FP), true positives (TP), false negatives (FN), and mAP at two different network sizes (320, 416) with IoU threshold 0.5, 0.75 and 0.5:0.95. To calculate precision and recall we use the TP, FP, and FN as shown in Eqs (8) and (9) whereas, F1-score is calculated by the resultant values of precision and recall as described in Eq (10). By summarizing the evaluation results based on the mAP, we can see that the model exhibited overall good performance, network size 416 with IoU threshold 0.5 have the highest mAP value of 97.84%. The Precision-recall curve (PR-curve) of COCO evaluation at the IoU threshold ranges from 0.5 to 0.95 at two network sizes is shown in Fig 6.

$$\begin{array}{c} Precision=\frac{TP}{TP+FP} \end{array}$$ (8)

$$\begin{array}{c} Recall=\frac{TP}{TP+FN} \end{array}$$ (9)

$$\begin{array}{c} F_{1}=2\times \frac{\text{Precision}\times \text{Recall}}{\text{Precision}+\text{Recall}} \end{array}$$ (10)

### Detection results

**Fig 7:**
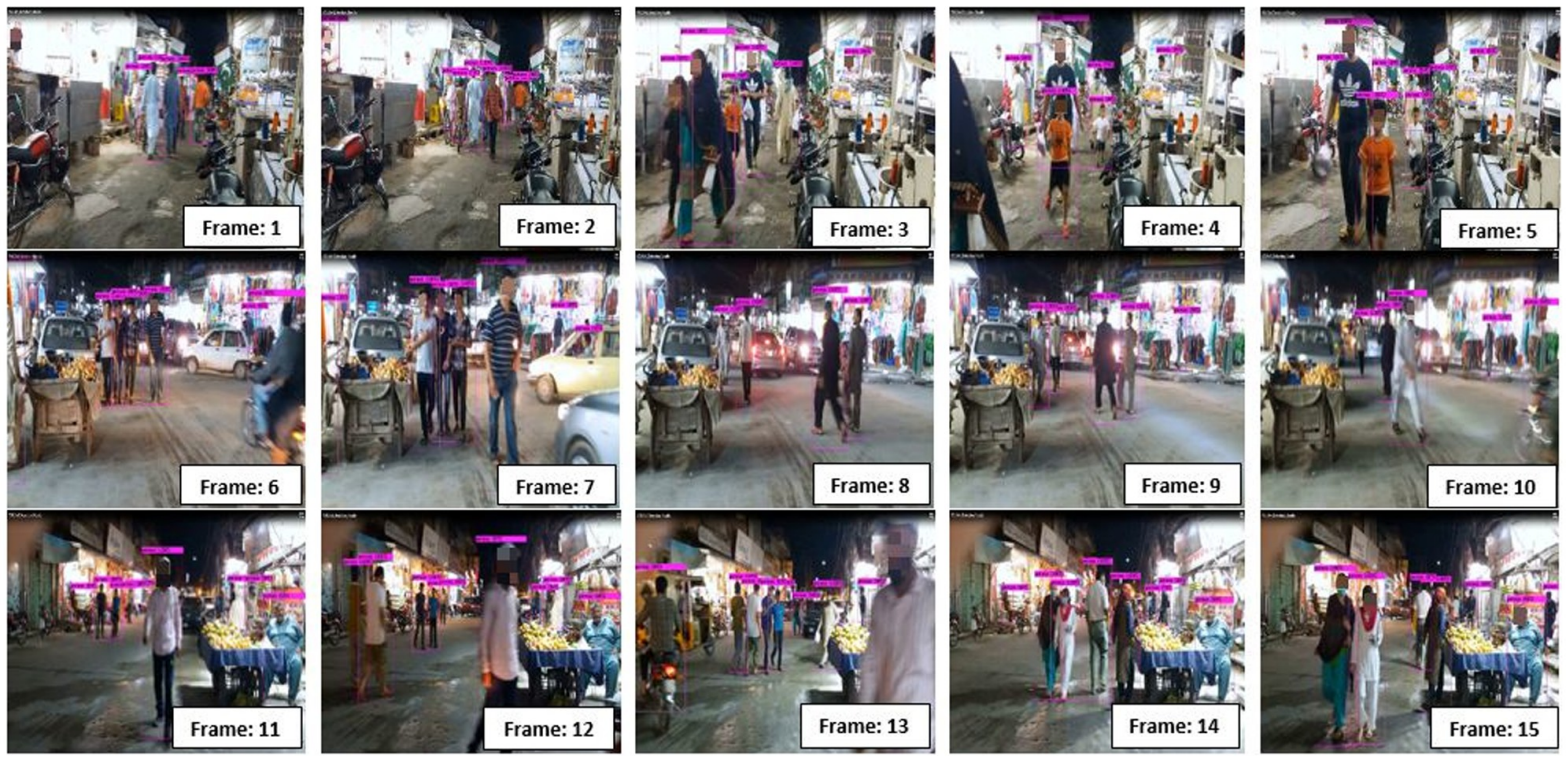
Visualization of classification and localization results of YOLO v4.

**Fig 8:**
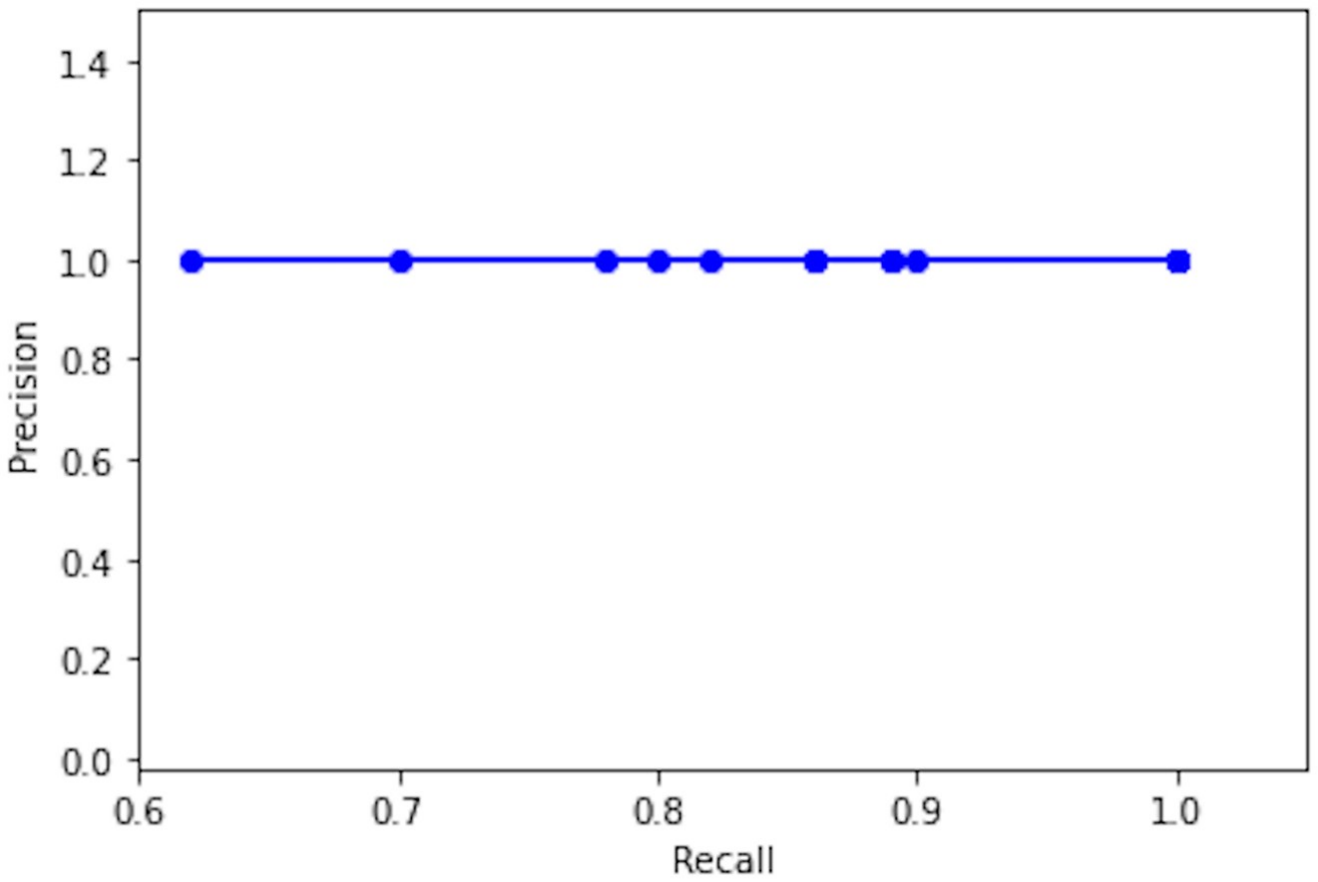
PR-curve for PR values of tested frames shown in Table 2.

We have tested our trained model on a custom dataset. Detection results per frame extracted from the video are shown in Fig 7. Table 2 shows TP, FP, FN, precision, and recall values for detected objects per frame. The model exhibited overall good performance in low light environments, from Table 2 it can be observed that no false positive is detected in any of the frames; whereas, the number of false-negatives is also low. PR-curve from precision-recall values of Table 2 is shown in Fig 8, we noticed that the precision values remained constant from Frame1 to Frame15.

**Table 2:** YOLO v4 performance evaluation results towards real-time person detection under various low light conditions from Fig 7.

Frames	TP	FP	FN	Prec.	Rec.
1	5	0	3	1	0.62
2	7	0	3	1	0.7
3	6	0	1	1	0.86
4	6	0	1	1	0.86
5	8	0	1	1	0.89
6	7	0	0	1	1
7	7	0	0	1	1
8	4	0	1	1	0.8
9	5	0	0	1	1
10	5	0	0	1	1
11	9	0	2	1	0.82
12	9	0	1	1	0.9
13	9	0	0	1	1
14	8	0	1	1	0.89
15	7	0	2	1	0.78

### Experimental results

**Fig 9:**
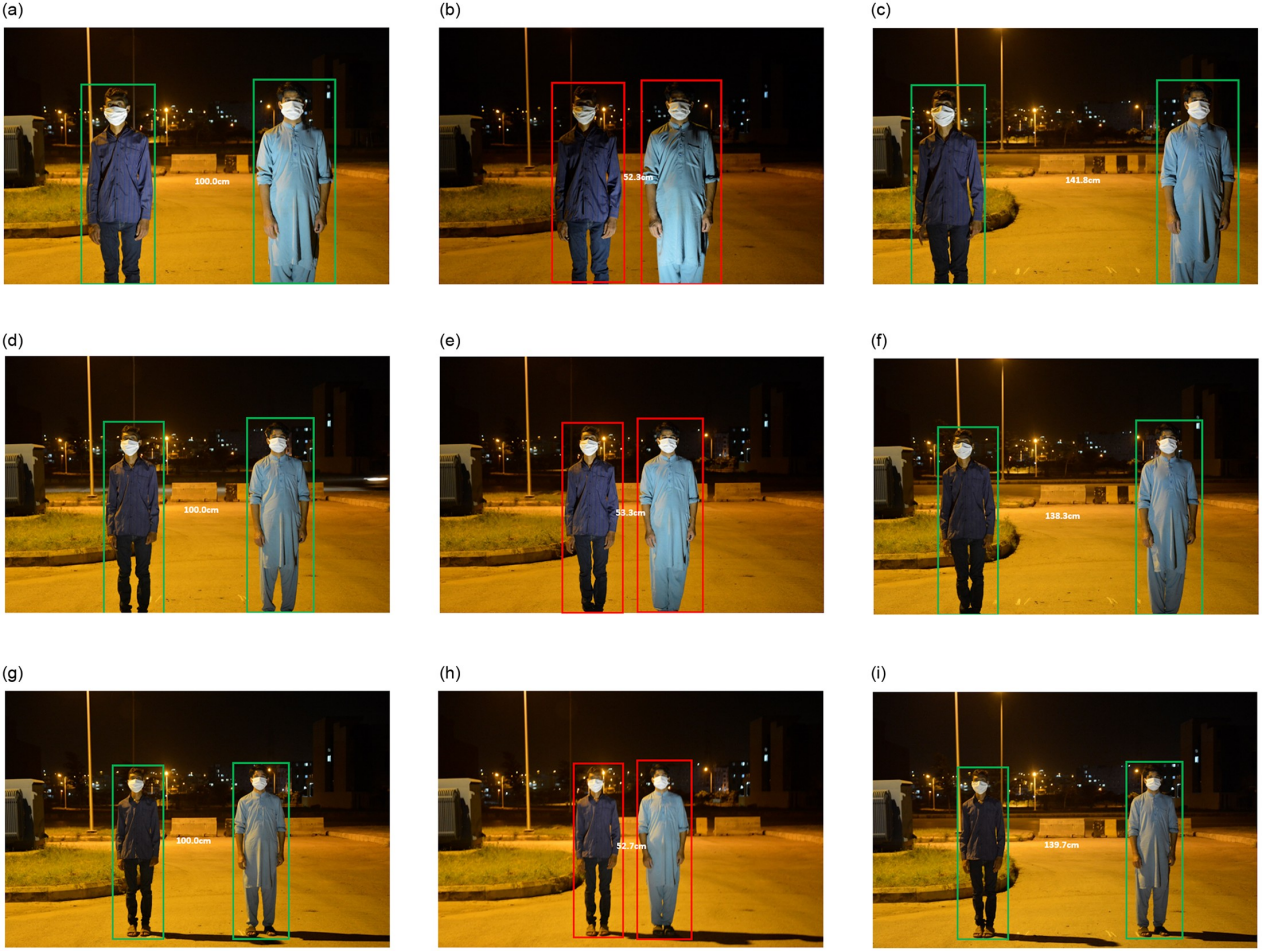
Test visualizations of our social distance monitoring approach at various *C*_*d*_ values. (a) *C_d_* = 400 cm (b) Test 1 (c) Test 2 (d) *C_d_* = 500 cm (e) Test 3 (f) Test 4 (g) *C_d_* = 600 cm (h) Test 5 (i) Test 6.

**Fig 10:**
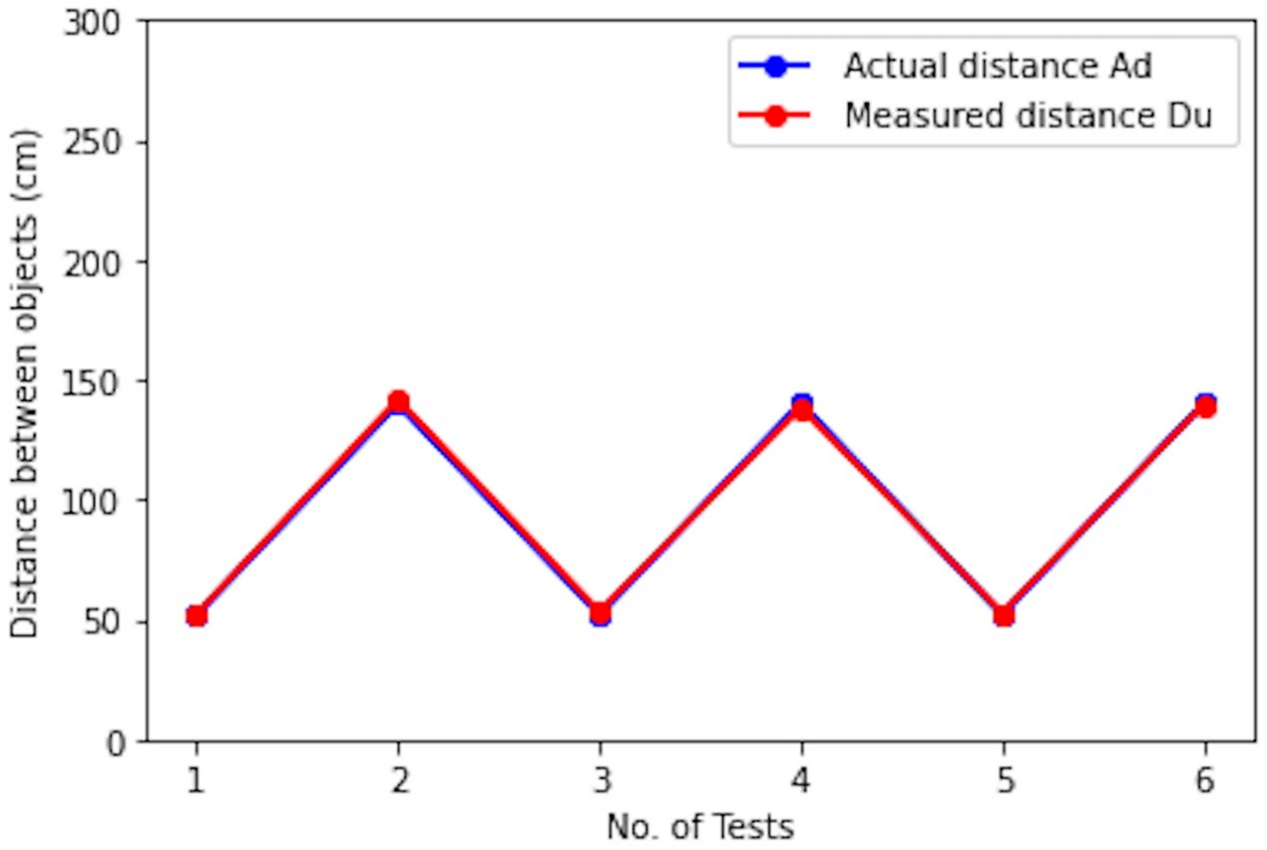
Graph plot of measured vs actual object distance values from Table 3 to highlight monitored error rate.

To evaluate the performance of our social distance monitoring solution, we perform few tests at three different fixed camera distances 400 cm, 500 cm, and 600 cm. Test frames are collected from the motionless ToF camera of Samsung galaxy note 10+ placed 4.5 feet above the ground where *C*_*p*_ is 0° (a regular camera view). At each specific fixed camera distance, we tested 2 scenarios one above the specified safety threshold (100 cm) at 140 cm and one below the specified safety threshold at 52 cm. Qualitative results are shown in Fig 9; whereas, Table 3 shows the quantitative results in terms of the distance between objects in pixels and cm, actual known distance in cm, and per test error rate. We can see that model exhibited overall good performance. People violating the safety distance are highlighted by red bounding boxes; whereas, green bounding boxes show people following safety distance criteria. The Absolute Error (AE) is calculated for all tests, between actual distance in units (*Ad*) and measured distance in units (*Du*) by using Eq (11) and based on AE mean absolute error (MAE) is calculated by Eq (12). The *Ad* and *Du* plot is shown in Fig 10, where the blue color shows the actual known distance in cm and the red line shows the measured distance in cm.

$$\begin{array}{c} AE=Du_{i}-Ad_{i} \end{array}$$ (11)

$$\begin{array}{c} MAE=\frac{1}{n}\sum_{i=1}^{n}\left|Du_{i}-Ad_{i}\right| \end{array}$$ (12)

**Table 3:** Social distancing measure tests at different *C*_*d*_ values as shown in Fig 10. Where *PD* is calculated distance in pixels, *Du* is measured distance in cm and *Ad* is actual distance in cm.

Test	*C*_*d*_	k	PD (pixels)	Du (cm)	Ad (cm)	AE (cm)
*D*_*T*1*T*2_ = 100 cm	400 cm	0.34236	292.098	-	-	-
1	-	-	153.0	52.3	52	0.3
2	-	-	414.1	141.8	140	1.8
*D*_*T*1*T*2_ = 100 cm	500 cm	0.40635	246.099	-	-	-
3	-	-	131.1	53.3	52	1.3
4	-	-	340.3	138.3	140	-1.7
*D*_*T*1*T*2_ = 100 cm	600 cm	0.49022	203.994	-	-	-
5	-	-	107.5	52.7	52	0.7
6	-	-	285.0	139.7	140	-0.3
**Mean Absolute Error (MAE) = 1.01 cm**						

## Limitations and discussion

This application is meant to be used in a real-time environment so, precision and accuracy are highly required to serve the motive. The proposed model shows efficient results during the evaluation of the YOLO v4 model in low light conditions where no single FP is detected, as the accuracy and reliability of the model is highly dependent on FP. To evaluate the performance of the social distance monitoring strategy few Tests are performed, as shown in Table 3. The proposed deep learning and motionless ToF camera-based social distance monitoring technique at *C*_*d*_ shows a good speed-accuracy tradeoff in monitoring social distancing during the night. The technique is limited to a few scenarios, social distance among people can be only monitored at fixed *C*_*d*_ values. Secondly, in order to initialize the monitoring process, we have to place two temporary target objects in an environment.

## Conclusion

This article proposes an efficient solution for real-time social distance monitoring in low light environments. For real-time person detection, the YOLO v4 algorithm is trained on the ExDARK dataset. For monitoring social distance, a motionless ToF camera is used to observe people at fixed camera distance and show resultant distance in real-world units. Safety distance violations are highlighted. The proposed YOLO v4 based real-time social distance monitoring solution is evaluated by COCO detection metrics. Experimental analysis shows that the YOLO v4 algorithm achieved the best results in different low light environments with 97.84% mAP score and the observed MAE value during the test of our social distance monitoring approach is 1.01 cm. The FPS score can be more enhanced by fine-tuning the same approach on GPUs like Volta, Tesla V100, or Titan Volta.

The proposed technique can be easily applied in real-world scenarios because of high precision and the low error rate, e.g., in banks to help the cashier to monitor people standing in front of him, in shops to help shopkeepers to observe customers, in train stations to help ticket giver to keep track of people violating safe distance, etc. In the future, we will extend our system to monitor social distance at varying camera distances by managing objects varying camera angles.
